# Severe Presentation of De Novo Type II Diabetes in Abiraterone and Prednisone Therapy

**DOI:** 10.7759/cureus.89841

**Published:** 2025-08-11

**Authors:** Martena Grace, Tanisha Vora, Amir A Estil-las, Camelia Arsene

**Affiliations:** 1 Medical School, Ross University School of Medicine, Bridgetown, BRB; 2 Internal Medicine, Trinity Health Oakland, Pontiac, USA

**Keywords:** abiraterone, abiraterone-induced hyperglycemia, diabetes, prednisone, prostate cancer, treatment-resistant prostate cancer

## Abstract

Abiraterone acetate is a widely used androgen biosynthesis inhibitor for the treatment of metastatic castration-resistant prostate cancer. It is usually co-administered with low-dose prednisone to counteract mineralocorticoid excess. While adverse effects such as hypertension, hypokalemia, and fluid retention are well recognized, hyperglycemia is a rare and underreported complication. We describe a case of a 77-year-old man with no prior history of diabetes who presented with acute severe symptomatic hyperglycemia after several months of treatment with abiraterone and low-dose prednisone. His laboratory workup revealed a serum glucose level of 431 mg/dL and hemoglobin A1c of 11.3%. The patient was managed with insulin therapy and later transitioned to metformin and basal insulin upon discharge. The temporal relationship between abiraterone initiation and the onset of dysglycemia raises concern for a potential causative link. Given the impact of hyperglycemia on cancer outcomes and quality of life, this case underscores the need for increased clinical awareness and routine glucose monitoring in patients treated with abiraterone and corticosteroids.

## Introduction

Abiraterone acetate is a medication used in the treatment of metastatic and castration-resistant prostate cancer [1,2]. After administration, it is metabolized to abiraterone, which inhibits the enzyme CYP17A1, a critical component of androgen biosynthesis. By blocking CYP17A1 activity, abiraterone effectively reduces androgen production, thereby limiting the growth and progression of prostate cancer [3,4]. CYP17AA1 inhibition also leads to an increase in mineralocorticoid levels, which may result in adverse effects such as hypertension, hypokalemia, and fluid retention. To mitigate these effects, low-dose prednisone is commonly co-administered, helping to suppress adrenocorticotropic hormone levels and reduce excess mineralocorticoids [5]. 

We present a rare case of a patient with stage IV prostate adenocarcinoma who initiated the standard dose of abiraterone in combination with low-dose prednisone. Shortly after completing one year of therapy, the patient developed an episode of severe and rapidly progressive hyperglycemia accompanied by episodes of ketoacidosis. This clinical course ultimately led to a new diagnosis of type 2 diabetes mellitus within a three-month span.

## Case presentation

We present the case of a 77-year-old man with a medical history of hypertension, hyperlipidemia, chronic obstructive pulmonary disease (COPD), chronic kidney disease (CKD), glaucoma, stage IIIa renal cell carcinoma, and stage IV prostate adenocarcinoma. The patient’s prostate cancer was diagnosed in February 2024, and he was started on abiraterone acetate 1,000 mg daily (four 250 mg tablets) in combination with prednisone 5 mg daily. 

The patient remained on this regimen until April 28, 2025, when he presented to the emergency department with a six-day history of progressive polyuria, polydipsia, generalized weakness, chills, and abdominal discomfort. He also reported decreased appetite and bilateral cold sensations in the lower extremities. Vital signs on presentation were unremarkable.

**Figure 1:**
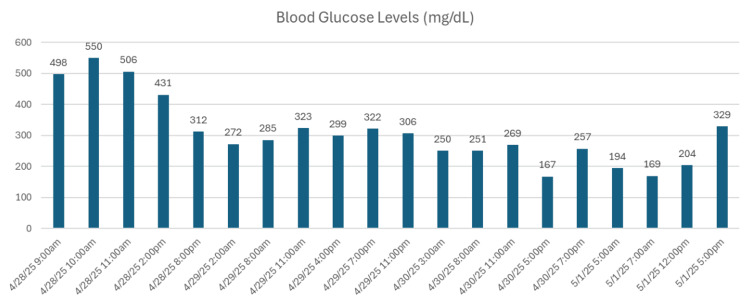
Blood Glucose Trend

Laboratory findings revealed a markedly elevated serum glucose level of 431 mg/dL and a hemoglobin A1c (HbA1c) of 11.3%. Serumlactate was mildly elevated at 2.1 mmol/L. Urinalysis demonstrated elevated beta-hydroxybutyrate levels, confirming ketosis. The patient was treated with intravenous hydration (normal saline), and insulin therapy was initiated with both glargine (Lantus) and insulin lispro. A trend of his blood glucose levels during hospitalization is shown in Figure 1.

Abiraterone was temporarily discontinued on April 28, 2025, during hospitalization and restarted on May 16, 2025, upon clinical stabilization. The patient was discharged on a new outpatient regimen that included metformin 500 mg twice daily and Lantus 30 units daily with a new diagnosis of type II diabetes mellitus. 

Interestingly, review of prior laboratory values showed that the patient’s HbA1c was within normal limits prior to initiation of abiraterone therapy (Table 1). This temporal association raises concern for abiraterone-associated dysglycemia, a phenomenon infrequently described in the literature.

**Table 1:** Patient's hemoglobin A1c (HbA1c)

Date	HbA1c
4/5/2022	6.1
10/21/2022	6
1/10/2025	6.4
4/28/2025	11.3

## Discussion

This case highlights a rare but clinically significant event in the setting of abiraterone acetate therapy: severe hyperglycemia in a patient with no prior history of diabetes mellitus. Although hyperglycemia is a recognized, yet infrequent, adverse effect of abiraterone, the development of rapid-onset and severe hyperglycemia progressing to diabetic ketoacidosis in an individual with no history of diabetes mellitus is exceedingly rare. Current literature review reveals only a handful of somewhat similar cases, with most lacking the acuity and severity as seen in our patient. 

As mentioned previously, abiraterone acetate is an inhibitor of CYP17A1, an enzyme critical for androgen synthesis. Its inhibition suppresses androgen production, thus delaying prostate cancer progression [2]. However, it also results in excess mineralocorticoid secretion and decreased cortisol levels, often necessitating co-administration of glucocorticoids such as prednisone. While prednisone-induced hyperglycemia is a recognized phenomenon, this patient’s acute metabolic decompensation occurred after months of stable therapy. This raises the question of whether abiraterone may have independently or synergistically contributed to worsening glycemic control. The study performed by Auchus et al. may hint at this synergistic effect. The authors claimed that mineralocorticoid-related adverse effects are rare with the current prednisone regimen the patient received, 5 to 10 mg daily [5]. Such adverse effects are expected to be seen in patients taking over 20 mg of prednisone daily, in the setting of malignancies or autoimmune illnesses [5]. Considering the unexpected incidence of hyperglycemia, the authors proposed evaluation of the patient’s ability to metabolize prednisone, assess comorbidities, and consider the length of androgen suppression, to help limit the possibility of hyperglycemia [5].

Although mineralocorticoid-related side effects of abiraterone such as hypertension and hypokalemia are well-established, reports of hyperglycemia remain limited [5]. A small number of observational studies and case reports have suggested impaired glucose tolerance in patients receiving long-term therapy, but the data remains inconclusive. A study by Goryachok et al. evaluated mineralocorticoid-related adverse effects in prostate cancer patients treated with abiraterone and either 5 mg or 10 mg of prednisone daily. Their findings revealed no statistically significant differences in body mass index (BMI) or rates of hyperglycemia between the two cohorts [6]. Similarly, a study by Fizazi et al. assessed corticosteroid-associated adverse events in patients on long-term, low-dose prednisone in combination with abiraterone. Consistent with the findings of Goryachok et al., they observed no significant increase in corticosteroid-associated complications, including hyperglycemia and weight gain [7].

While the direct association between abiraterone and the development of hyperglycemia or diabetes mellitus remains uncertain, the presence of dysglycemia in this population carries important clinical implications. Diabetes mellitus has been shown to negatively impact survival in patients with prostate cancer. In a retrospective analysis by Karantanos et al., 61 patients with castration-resistant prostate cancer treated with abiraterone or enzalutamide, another anit-androgen medication, were evaluated. The study demonstrated that patients with well-controlled glycemic indices (HbA1c 4.7-5.9%) had a significantly longer median survival (6.72 ± 1.3 months; p<0.0001) compared to those with poorly controlled diabetes (HbA1c 7.8-11.6%) [8].

Considering that our patient is now a type 2 diabetic, his antihyperglycemic medications are being adjusted to optimally control blood glucose levels. Interestingly, Tucci et al. presented two cases in which patients with metastatic castration-resistant prostate cancer and diabetes mellitus taking abiraterone, sulphonylureas, statins, and prednisone resulted in severe episodes of hypoglycemia [9]. The authors concluded that drug-drug interactions between abiraterone, statins, and repaglinide, a sulphonylurea, may be responsible for the episode of hypoglycemia. Repaglinide is metabolized by CYP2C8 and CYP3A4. Simvastatin is a known inhibitor of CYP3A4, which increases the bioavailability of repaglinide. The patient in the case report experienced severe hypoglycemia, for which abiraterone was suspended and he was maintained on repaglinide. The blood glucose levels were maintained within normal limits until abiraterone was reintroduced with a 50% reduction in repaglinide dosing, resulting in another episode of severe hypoglycemia [9]. This unique presentation warrants further study, but serves as an example to maintain strict glucose control and monitoring in diabetic patients taking abiraterone and prednisone. 

Although this case represents a rare and interesting clinical finding, it remains a single-patient observation. Therefore, no definitive conclusions can be drawn regarding causality. Nonetheless, this case underscores a potential metabolic complication of abiraterone-prednisone therapy that may be underrecognized. It also raises the important questions about whether routine glucose monitoring should be considered in patients initiating this regimen, particularly those with predisposing risk factors for dysglycemia. Further research is warranted to clarify the incidence, mechanism, and long-term implications of treatment-associated hyperglycemia in this population.

## Conclusions

This case highlights a rare but potentially significant complication of abiraterone and prednisone therapy: severe hyperglycemia in a patient without prior history of diabetes. Although a direct causal relationship cannot be definitively established from a single case, the temporal and sudden association and absence of alternative explanations raise concern for a possible drug-induced effect. Given the increasing use of abiraterone in the management of advanced prostate cancer, clinicians should remain vigilant for new-onset hyperglycemia, among other metabolic disturbances. Routine monitoring of glucose levels may be warranted, particularly in patients with existing risk factors for impaired glucose tolerance. Further studies are needed to better characterize the incidence, mechanisms and management of this potential adverse effect.
